# Defect Engineering of Nanomaterials for Catalysis

**DOI:** 10.3390/nano13061116

**Published:** 2023-03-21

**Authors:** Yang Luo, Yinghong Wu

**Affiliations:** 1 Department of Materials, ETH Zürich, Zürich 8093, Switzerland; 2 Department of Physics, City University of Hong Kong, Kowloon 999077, Hong Kong SAR, China; 3 Department of Health Sciences and Technology, ETH Zürich, Zürich 8008, Switzerland

## 1. Introduction

Defect chemistry is a branch of materials science that deals with the study of the properties and behavior of defects in crystalline solids [1]. Defects in materials can arise from various sources, including manufacturing processes, environmental factors, and intrinsic properties of the material. Defects can have a significant impact on the physical and chemical properties of materials, including their mechanical, optical, electrical, and especially catalytic properties.

## 2. Defective Catalysts

**Figure 1:**
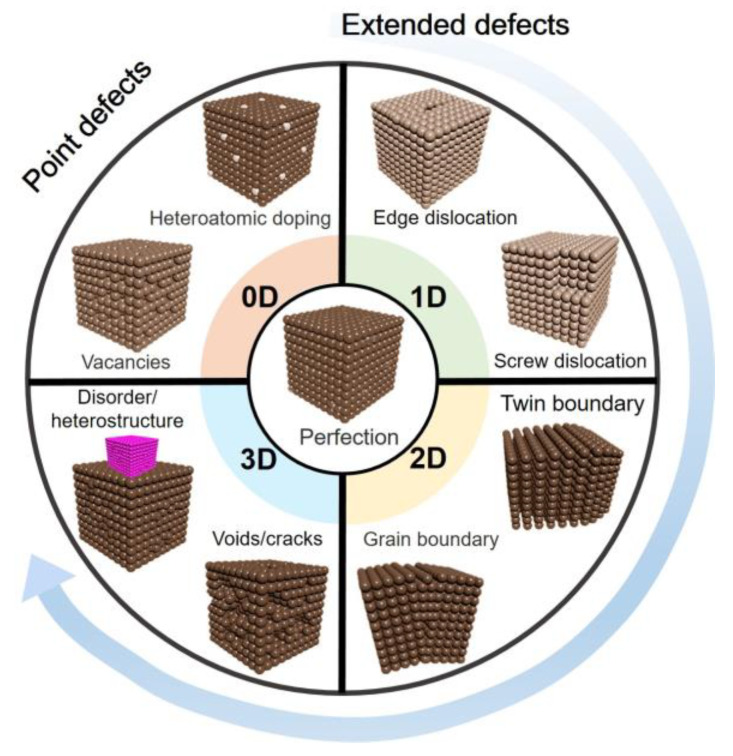
Types of defects in materials [3].

Defects in materials can be classified into two main categories: point defects and extended defects [2], as summarized in Figure 1. Point defects are localized defects that occur at a single lattice site in the crystal structure. Point defects can be further classified into three types: vacancy defects, interstitial defects, and substitutional defects. Vacancy defects occur when an atom is missing from its lattice site, creating a vacancy in the crystal structure. Interstitial defects occur when an atom occupies a site between lattice points. Substitutional defects occur when one atom is replaced by another atom of a different element. Extended defects are defects that occur over a larger area of the crystal structure. Extended defects can be further classified into two types: dislocations and grain boundaries. Dislocations are regions in the crystal where the lattice structure is distorted, while grain boundaries are regions where two grains with different crystal orientations contact.

Defects play a crucial role in catalysis because they can significantly alter the surface reactivity of materials. In catalysis, reactants bind to the surface of a catalyst, where they undergo chemical transformations that lead to the formation of products. In general, defects on the surface of a catalyst can enhance the reactivity by providing active sites for reactants to bind to and undergo chemical reactions. This is because defects can create unsaturated coordination sites, which are more reactive than fully coordinated sites. For example, a metal oxide surface with a missing atom, vacancy defect, can act as a strong adsorption site for reactant molecules [4,5]. Defects can also modify the electronic structure of a catalyst, leading to changes in its catalytic activity. For example, substitutional defects, where one atom is replaced by another, can introduce new electronic states in the catalyst that can affect its reactivity towards specific reactants [6,7]. Similarly, dislocations and grain boundaries can create regions with different electronic properties, leading to increased catalytic activity in these regions [8,9]. Therefore, understanding the role of defects in catalysis is important for designing catalysts with improved activity, selectivity, and durability. Defect engineering is an emerging field in catalysis that focuses on regulating the properties and distribution of defects in materials to achieve desired catalytic properties. This strategy involves intentionally introducing defects in catalysts or modifying their defect structures to optimize their catalytic activity. By tailoring the defect structures of materials, it is possible to improve their catalytic properties and prepare more efficient and sustainable catalysts for a wide range of applications such as energy conversion and storage, chemical production, and environmental remediation.

## 3. Preparation Strategies for Defective Catalysts

There are several methods to prepare defects on catalysts, and the choice of method depends on the type of defect and the specific catalyst adopted. Researchers have demonstrated some commonly used methods for preparing defects on catalysts. Annealing involves heating the catalyst at high temperatures under controlled conditions to induce defects. For example, annealing a metal catalyst in a reducing atmosphere can create surface vacancies that can act as active sites for catalytic reactions [10,11]. Ion implantation involves introducing ions of a specific element into the catalyst material to create substitutional defects. For example, selected ions can be accelerated to a high energy and then implanted into a material’s surface to generate a higher surface area and a higher concentration of active sites, which could significantly enhance the catalytic activity for a variety of chemical reactions [12,13]. This method allows for precise control of the location and concentration of defects in the catalyst. Chemical etching involves treating the catalyst with a chemical solution to remove atoms from the surface of the material, creating defects such as vacancies and step edges. It is a simple and low-cost technique that can be easily applied to a wide range of catalysts [14,15]. Mechanical treatment involves physically grinding or polishing the catalyst material to create defects such as dislocations and grain boundaries. For example, high-energy ball milling is suitable to create a high density of dislocations, vacancies, and other defects on catalysts [16,17]. Plasma treatment involves exposing the catalyst material to a plasma source, which can create defects such as surface vacancies and heteroatomic doping [3]. Electrochemical treatment involves applying a voltage or current to the catalyst material in a liquid electrolyte solution to induce surface reactions and create defects. The biggest advantage of the electrochemical method is the selectively removing or depositing atoms or molecules from the catalyst surface in a mild environment [18,19].

It Is important to note that the preparation of defects on catalysts requires careful control of the reaction conditions, such as temperature, pressure, and exposure time, to ensure that the resulting defects are stable and reproducible. Furthermore, the type and distribution of defects created can strongly affect the catalytic activity and selectivity of the catalyst, so it is important to carefully characterize the defects using techniques such as microscopy, spectroscopy, and surface analysis to fully understand their properties and behavior.

## 4. Characterization of Defects

Scanning Electron Microscopy (SEM) can be used to visualize the surface of the catalyst and identify structural defects such as cracks, pits, and grain boundaries. Transmission Electron Microscopy (TEM) can be used to visualize the crystal structure of the catalyst at the atomic scale to detect structural defects such as dislocations, stacking faults, and grain boundaries. X-ray Photoelectron Spectroscopy (XPS) can detect chemical defects such as vacancies, surface oxides, and impurities. Raman Spectroscopy can be used to analyze the vibrational modes of the catalyst material and detect defects such as carbonaceous species and surface contaminants. Fourier Transform Infrared Spectroscopy (FTIR) can be used to analyze the vibrational modes of molecules on the catalyst surface and detect adsorbed species such as CO, NO, and H_2_O. X-ray Absorption Spectroscopy (XAS) can detect structural defects such as edge sites, vacancies, and impurities by analyzing the local structure and electronic properties of the catalyst material. Atomic Force Microscopy (AFM) can detect surface defects such as step edges and vacancies. Thermal Analysis techniques such as thermogravimetric analysis (TGA) and differential scanning calorimetry (DSC) can be used to analyze the thermal stability and reactivity of the defective catalyst material with surface oxides, carbonaceous species, and other adsorbed species. By using these characterization techniques in combination, it is possible to gain a detailed understanding of the structural, chemical, and electronic properties of the catalyst material, which can help to identify and optimize the defect structures for specific catalytic applications.

## 5. Stability and Dynamic Evolution of Defective Catalysts

The stability of defective catalysts depends on the type and distribution of defects in the material, as well as the operating conditions of the catalytic reaction. In general, defects can have both positive and negative effects on the stability of catalysts. On the one hand, defects can increase the catalytic activity of a material by creating active sites for reactant adsorption and reaction. However, defects can also lead to the formation of unwanted reaction intermediates or byproducts, which can accumulate on the surface of the catalyst and reduce its activity over time. For example, carbon deposition on the surface of metal catalysts can lead to deactivation and loss of activity over time [20,21]. In addition, defects can affect the stability of the catalyst by altering its surface energy and reactivity. For example, vacancies and step edges can have higher surface energy than fully coordinated sites, making them more prone to surface oxidation and corrosion. Substitutional defects can also introduce new electronic states that can alter the reactivity and stability of the catalyst. Although defects usually atomic defects are unstable in most cases, the developed defective catalyst can show high performance in the very long term. This is closely related to the reconstruction and dynamic evolution of defects in electrochemical reactions.

Operando characterization involves studying the catalytic reaction under actual operating conditions to gain insight into the stability and dynamic evolution behavior of the catalyst in real time. In situ X-ray diffraction (XRD) can track the evolution of defects such as lattice strain and lattice expansion by monitoring the changes in the diffraction patterns in real time during electrochemical reactions. By monitoring the changes in the in situ XAS spectra, it is possible to track the evolution of defects such as the oxidation state of the catalyst and the formation of surface species. In situ scanning tunneling microscopy (STM) observes the changes in the surface structure and morphology to track the evolution of defects such as the formation of surface vacancies and the migration of surface species. In situ Raman spectroscopy can be used to monitor the evolution of defects such as the formation of surface oxides and the adsorption of surface species. In situ electrochemical impedance spectroscopy (EIS) can track the evolution of defects such as changes in the conductivity and charge transfer kinetics of the catalyst material. By combining these in situ techniques, it is possible to gain a detailed understanding of the evolution of defects during electrochemical reactions, which can help to optimize the design and performance of electrochemical devices for a wide range of applications.

## 6. Challenges and Visions

**Figure 2:**
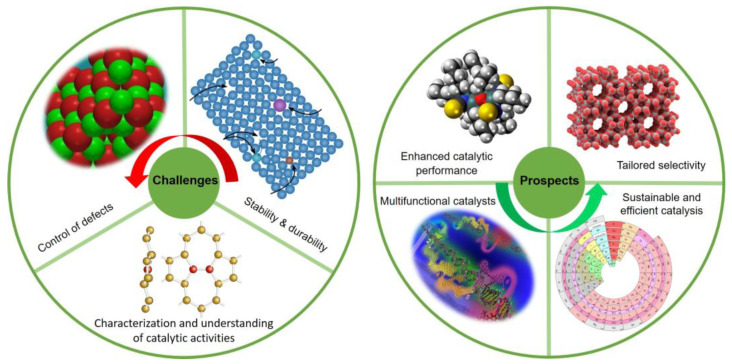
Challenges and prospects for defective catalysts.

This Special Issue aims to provide a platform for researchers to share their latest findings and discuss current trends in the development and characterization of defective nanocatalysts. Defective catalysts have been widely studied in various fields due to their unique properties and potential applications, as summarized in the following. Energy conversion and storage: Defective catalysts have shown great potential in the fields of electrocatalysis and photocatalysis for energy conversion and storage applications. For example, defective metal oxide nanoparticles and transition metal chalcogenides have been reported to exhibit enhanced electrocatalytic activity for hydrogen evolution reaction (HER), oxygen evolution reaction (OER) and oxygen reduction reaction (ORR) [22,23,24]. Environmental remediation: Defective catalysts have also been extensively studied for environmental remediation applications, such as air and water purification. For example, defective TiO_2_ nanoparticles have been reported to exhibit enhanced photocatalytic activity for the degradation of organic pollutants [25], and defective metal-organic frameworks (MOFs) have been shown to be effective adsorbents for heavy metal ion removal [26]. Chemical synthesis: Defective catalysts have also shown promising results in chemical synthesis and reaction engineering applications, such as selective catalytic reduction (SCR) of NO_x_ and olefin polymerization. For example, defective zeolites have been reported to exhibit higher selectivity and activity in the SCR of NO_x_ compared to their defect-free counterparts [27], and defective graphene oxide has been demonstrated to be a highly efficient catalyst for olefin hydrogenation [28]. Biomedical applications: Defective catalysts have recently gained attention for their potential applications in the biomedical field. For example, defective metal nanoparticles have been reported to exhibit enhanced photothermal therapy (PTT) efficacy for cancer treatment [29], and defective metal–organic frameworks have been shown to be promising drug delivery vehicles due to their tunable pore sizes and surface functionalization [30]. Overall, defective catalysts have shown great potential in a wide range of fields and continue to be an active area of research. The development of defective catalysts presents both challenges and prospects as summarized in Figure 2.

### 6.1. Challenges

- Control of defects: One of the main challenges in the development of defective catalysts is the precise control of the type, density, and distribution of defects. The preparation of defective catalysts often involves complex synthesis methods that can result in unwanted defects or inconsistent properties;
- Stability and durability: Defective catalysts may be more active, but they are also more prone to deactivation and instability due to the presence of defects. Ensuring the stability and durability of defective catalysts remains a significant challenge;
- Characterization and understanding of catalytic activities: The characterization of defective catalysts can be challenging, as their properties may differ significantly from those of their defect-free counterparts. Moreover, the understanding of the underlying mechanisms that govern the enhanced activity and selectivity of defective catalysts is still incomplete.

### 6.2. Prospects

- Enhanced catalytic performance: The primary prospect of developing defective catalysts is their potential to exhibit enhanced catalytic performance compared to their defect-free counterparts. The presence of defects can introduce new active sites, alter the electronic and structural properties of the catalyst, and modify the adsorption and diffusion properties of reactant molecules;
- Tailored selectivity: Defective catalysts can also exhibit tailored selectivity towards specific products, making them highly valuable for fine chemical synthesis and other applications;
- Multifunctional catalysts: The unique properties of defective catalysts can enable them to serve as multifunctional catalysts, simultaneously catalyzing multiple reactions or performing other functions such as sensing and imaging;
- Sustainable and efficient catalysis: Defective catalysts may also offer a promising route to sustainable and efficient catalysis, by reducing the need for expensive or toxic co-catalysts, and enabling the use of renewable energy sources for catalytic reactions;
- The guest editor hopes that by understanding the properties and behavior of defects in materials, scientists and engineers can design materials with improved catalytic activities, as well as develop new applications for these materials.

## Data Availability

Data sharing is not applicable to this article.
